# Sepsis-Associated DIC with Decreased Levels of Antithrombin and Fibrinogen is the Target for Combination Therapy with Thrombomodulin Alfa and Antithrombin

**DOI:** 10.1055/a-2009-9073

**Published:** 2023-02-22

**Authors:** Hideo Wada, Kazuo Kawasugi, Goichi Honda, Noriaki Kawano, Toshimasa Uchiyama, Seiji Madoiwa, Naoki Takezako, Kei Suzuki, Yoshinobu Seki, Takayuki Ikezoe, Toshiaki Iba, Kohji Okamoto

**Affiliations:** 1 Department of General Medicine, Mie Prefectural General Medical Center, Mie, Japan; 2 Faculty of Medical Technology, Teikyo University, Tokyo, Japan; 3 Department of Medical Affairs, Asahi Kasei Pharma Corporation, Tokyo, Japan; 4 Department of Internal Medicine, Miyazaki Prefectural Miyazaki Hospital, Miyazaki, Japan; 5 Department of Laboratory Medicine, National Hospital Organization Takasaki General Medical Center, Gunma, Japan; 6 Department of Clinical Laboratory Medicine, Tokyo Saiseikai Central Hospital, Tokyo, Japan; 7 Department of Hematology, Nerima Hikarigaoka Hospital, Tokyo, Japan; 8 Emergency and Critical Care Center, Mie University Hospital, Mie, Japan; 9 Department of Hematology, Uonuma Institute of Community Medicine, Niigata University Medical and Dental Hospital, Niigata, Japan; 10 Department of Hematology, Fukushima Medical University, Fukushima, Japan; 11 Department of Emergency and Disaster Medicine, Juntendo University Graduate School of Medicine, Tokyo, Japan; 12 Department of Surgery, Center for Gastroenterology and Liver Disease, Kitakyushu City Yahata Hospital, Fukuoka, Japan

**Keywords:** thrombomodulin, antithrombin, fibrinogen, disseminated intravascular coagulation, sepsis

## Abstract

**Background** Disseminated intravascular coagulation (DIC) is not a homogeneous condition, but rather includes heterogeneous conditions, and its pathophysiology and outcome vary considerably depending on the background. Although anticoagulant therapy is expected to be of benefit in the treatment of DIC, previous studies have suggested that the benefits are limited only to a specific subtype.

**Objects** The purpose of this study was to identify the group that would benefit from combination therapy using thrombomodulin/antithrombin.

**Methods** The data from 2,839 patients registered in the postmarketing surveillance of thrombomodulin were evaluated. The patients were divided into four groups depending on antithrombin and fibrinogen levels, and the additive effects of antithrombin on thrombomodulin were examined in the groups.

**Results** The DIC score, Sequential Organ Failure Assessment score, and mortality were significantly higher in the DIC group with low-antithrombin/low-fibrinogen than in the DIC groups without either low antithrombin or low fibrinogen. The survival curve was significantly higher in DIC patients with combination therapy than in patients treated with thrombomodulin monotherapy, but this effect was seen only in patients with infection-based DIC.

**Conclusion** DIC patients with low-antithrombin/low-fibrinogen risk poor outcomes, but they can be the target of combination therapy with antithrombin and thrombomodulin as long as the DIC is due to infection.

## Graphical abstract

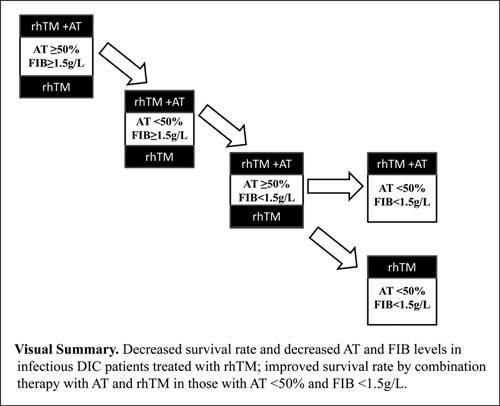

## Introduction

Disseminated intravascular coagulation (DIC) is a critical condition that is frequently associated with various diseases, including infectious diseases, multiple trauma, solid cancers, and hematologic malignancy. Severe life-threatening bleeding and/or organ failure are typical features of the advanced stage of DIC, commonly resulting in poor outcomes. 1 2 3 The systemic activation of coagulation followed by consumptive coagulopathy is reflected by the increased fibrin generation and decreased activated coagulation inhibitors such as antithrombin (AT), protein C, protein S, and thrombomodulin (TM). Decreases in hemostatic factors, such as fibrinogen and platelet count, are also known as the hallmarks of DIC. 3 4 5

Four diagnostic criteria are commonly used clinically: the Japanese Ministry of Health, Labor and Welfare (JMHLW) criteria, 6 the International Society of Thrombosis Haemostasis (ISTH) criteria, 4 the Japanese Association for Acute Medicine (JAAM) criteria, 7 and the Japanese Society of Thrombosis and Hemostasis (JSTH) criteria. 8 All of these criteria adopted a scoring system calculated with similar laboratory tests, that is, platelet count, fibrinogen, prothrombin time (PT), and fibrin degradation products (FDPs). 4 6 7 8 It has been realized that the characteristics of DIC vary significantly depending on the underlying conditions, and the JAAM criteria eliminated fibrinogen because the target was restricted to acute DIC. Meanwhile, the JMHLW and JSTH divided the scoring system into subclasses depending on the underlying diseases. 8 9

With respect to management, the British Committee for Standards in Haematology, the JSTH, the Italian Society for Thrombosis and Haemostasis, and the ISTH have established guidelines for the diagnosis and treatment of DIC. 10 11 12 13 Management of the underlying diseases is the common recommendation, but the recommendation for anticoagulation is inconsistent. For example, administration of AT and TM is recommended only in the JSTH guidelines. 11 14 Since multiple randomized, controlled studies and their post hoc analyses and other clinical studies have shown the potential efficacy of TM, 15 16 17 18 TM is widely used for infectious and hematological DIC in Japan. In contrast, there has not been a large-scale, randomized, controlled trial that examined the effect of AT or activated protein C on DIC. 19 20 21 Since the 1980s, AT has been commonly used for the treatment of DIC patients with decreased AT levels in Japan.

Decreased AT levels and hypofibrinogenemia have been shown to independently predict poor outcomes in postmarketing surveillance (PMS). 22 23 In the present study, DIC was classified by AT and fibrinogen levels to examine their usefulness as severity markers. Then, the usefulness of the categorization based on AT and fibrinogen levels to select the target of combination therapy of AT and TM was also evaluated.

## Methods

### Study Design and Data Collection

The original PMS study was an open-label, multicenter, noninterventional, prospective, observational cohort study of patients with DIC who received recombinant soluble TM (TM-α; 2008–2010). 16 The PMS for TM-α was conducted in accordance with the JSTH Post-Marketing Surveillance Committee for TM-α injection and the guidelines for Good Post-Marketing Surveillance Practices, as required by the JMHLW. Existing data without personally identifiable information were used throughout the study. The original PMS study was therefore exempted from local institutional review and formal approval, as well as the requirement for informed consent. All patients who received TM-α were consecutively registered on initiation of treatment by documenting the patients' demographics using a central registration system. The patients were prospectively observed until 28 days after administration of TM-α. The standard dose of TM-α was 380 U/kg, and the adjusted dose of 130 U/kg was used for patients with renal dysfunction. All patients were treated according to the attending physician's decisions, and there was no limitation on the concomitant use of other anticoagulants or medicine for the treatment of underlying diseases and complications. The PMS study collected 4,260 case reports over a period of approximately 2 years.

**Fig. 1:**
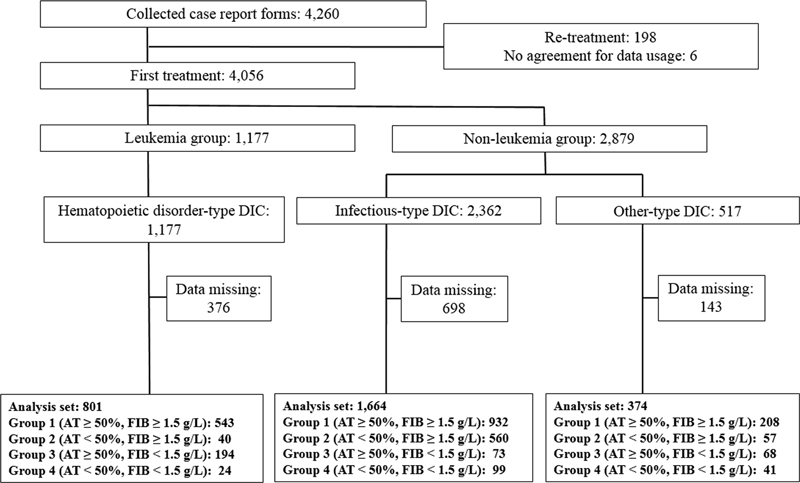
Classification of DIC in postmarketing surveillance data using antithrombin and fibrinogen levels. DIC, disseminated intravascular coagulation; AT, antithrombin; FIB, fibrinogen.

A post hoc analysis of the PMS data of TM-α was conducted. Of the 4,260 patients, the 4,056 patients who underwent first TM-α administration were divided into three groups (hematopoietic disorder-type, infectious-type, and other-type) on the basis of the underlying disease in accordance with the JSTH DIC definition. 8 Furthermore, each underlying disease type group was divided into four groups according to the combination of baseline AT and fibrinogen levels: group 1 (AT ≥ 50%, fibrinogen ≥ 1.5 g/L); group 2 (AT < 50%, fibrinogen ≥ 1.5 g/L); group 3 (AT ≥ 50%, fibrinogen < 1.5 g/L); and group 4 (AT < 50%, fibrinogen < 1.5 g/L) ( Fig. 1 ).

### Evaluation

The analyses included 2,839 DIC patients (infectious-type, *n* = 1,664; hematopoietic disorder-type, *n* = 801; other-type, *n* = 374) from the PMS of TM-α. The primary objective of the present study was to compare the 28-day survival curves of the four groups divided according to baseline fibrinogen and AT levels by underlying disease type. The secondary objective was to investigate the outcomes, including the clinical features of DIC patients, DIC resolution rates, 28-day survival rates, and subgroup analysis with and without AT therapy, among the DIC patients.

The degree of coagulopathy was evaluated by calculating DIC scores according to the DIC diagnostic criteria of the JAAM 7 for infectious-type and other-type DIC and those of the JMHLW 6 and ISTH 4 for hematopoietic disorder-type, infectious-type, and other-type DIC. After treatment with TM-α, resolution of DIC was defined as a score ≤ 3 using the diagnostic criteria of the JAAM, ≤ 2 using those of the JMHLW for DIC in patients with hematopoietic disorder-type, ≤ 5 using those of the JMHLW for infectious-type and other-type DIC, and ≤ 4 using those of the ISTH for all types.

In infectious-type or other-type DIC, the severity of organ failure was assessed using the Sequential Organ Failure Assessment (SOFA) score. 24 Positive symptoms for organ failure were determined by the attending physician based on clinical signs indicating organ dysfunction due to DIC. 6 Laboratory tests such as the platelet count and hemostatic tests such as the PT ratio, fibrinogen and FDPs, AT, thrombin-AT complex (TAT), and plasmin-α2 plasmin inhibitor complex were measured in each participating institute.

### Statistical Analysis

Data are expressed as numbers (%) or medians (quartiles [Q1, Q3]). The baseline demographics of each group were compared with group 1 as the control using nonparametric multiple testing. Serial changes in the clinical data of each group (groups 1–4) were compared using the Wilcoxon signed-rank test, as appropriate. The Kaplan–Meier method and log-rank test were used to assess survival. *p* -Values of < 0.05 were considered significant. Multiplicity adjustment was not considered. All analyses were performed using SAS version 9.4 (SAS Institute, Cary, North Carolina, United States) by EPS Corporation (Tokyo, Japan) according to the statistical analysis plan.

## Results

The sites of infection for the infectious-type and the underlying disease for the hematopoietic disorder-type are listed in Table 1 . Other-type included patients with solid tumors, pancreatitis, shock, and burns.

**Table 1:** Underlying disease types

Underlying disease type	Group 1 AT ≥ 50% FIB ≥ 1.5 g/L	Group 2 AT < 50% FIB ≥ 1.5 g/L	Group 3 AT ≥ 50% FIB < 1.5 g/L	Group 4 AT < 50% FIB < 1.5 g/L
Infectious diseases; site of infection, *n* (%)				
Respiratory	220 (23.6)	123 (22.0)	19 (26.0)	25 (25.3)
Abdominal	141 (15.1)	132 (23.6)	9 (12.3)	27 (27.3)
Urinary or genital	117 (12.6)	51 (9.1)	7 (12.3)	6 (6.1)
Hepatobiliary or pancreatic	71 (7.6)	55 (9.8)	6 (8.2)	8 (8.1)
Surgical site, soft tissue, or bone	37 (4.0)	36 (6.4)	2 (2.7)	5 (5.1)
Central nervous system	35 (3.8)	6 (1.1)	3 (4.1)	2 (2.0)
Cardiovascular	15 (1.6)	6 (1.1)	5 (6.8)	1 (1.0)
Blood stream	286 (30.7)	146 (26.1)	21 (28.8)	25 (25.3)
Others	10 (1.1)	5 (0.9)	1 (1.4)	0 (0.0)
Hematological diseases, *n* (%)				
Acute myeloid leukemia	173 (31.9)	6 (15.0)	41 (21.1)	2 (8.3)
Acute promyelocytic leukemia	53 (9.8)	0 (0.0)	69 (35.6)	1 (4.2)
Acute lymphocytic leukemia	71 (13.1)	0 (0.0)	28 (14.4)	3 (12.5)
Chronic myelogenous leukemia	11 (2.0)	0 (0.0)	1 (0.5)	0 (0.0)
Chronic lymphocytic leukemia	3 (0.6)	0 (0.0)	1 (0.5)	2 (8.3)
Myelodysplastic syndromes	24 (4.4)	3 (7.5)	4 (2.1)	1 (4.2)
Multiple myeloma	12 (2.2)	3 (7.5)	3 (1.5)	1 (4.2)
Lymphoma	65 (12.0)	13 (32.5)	31 (16.0)	8 (33.3)
Others	10 (1.8)	2 (5.0)	7 (3.6)	3 (12.5)
Unknown	121 (22.3)	13 (32.5)	9 (4.6)	3 (12.5)

Abbreviations: AT, antithrombin; FIB, fibrinogen.

Note: Data for treatment are shown as numbers (%).

The frequency of group 1 was 56.0, 67.8, and 55.6% in infectious-type, hematopoietic disorder-type, and other-type DIC, respectively. That of group 2 was 33.7, 5.0, and 15.2% in infectious-type, hematopoietic disorder-type, and other-type DIC, that of group 3 was 4.4, 24.2, and 18.2%, and that of group 4 was 5.9, 3.0, and 11.0%, respectively ( Table 2 ). The frequency of renal and liver dysfunction tended to be higher in groups 2 and 4, and the frequency of hemorrhage tended to be higher in group 3. The SOFA score of infectious-type and other-type of DIC was significantly higher in groups 2 and 4 than in group 1 (infectious-type: groups 2, 4 *p* < 0.001, other-type: groups 2, 4 *p* < 0.01). The JAAM DIC score of infectious-type DIC was significantly higher in groups 2, 3, or 4 than in group 1 (group 2 *p* < 0.001, group 3 *p* < 0.01, group 4 *p* < 0.05). ISTH overt-DIC and JMHLW DIC scores of all types of DIC were significantly higher in group 3 or 4 than in group 1 (ISTH overt-DIC scores, hematopoietic disorder-type: group 3 *p* < 0.001, other-type: groups 3, 4 *p* < 0.001, infectious-type: group 3 *p* < 0.01, infectious-type: group 4 *p* <0.05, hematopoietic disorder-type: group 4 *p* < 0.05, JMHLW DIC scores, all *p* < 0.001). These patients with DIC were managed with several interventions, as shown in Table 2 .

**Table 2:** Subjects by group and DIC type

	Type of DIC	Group 1 AT ≥ 50% FIB ≥ 1.5 g/L	Group 2 AT < 50% FIB ≥ 1.5 g/L	Group 3 AT ≥ 50% FIB < 1.5 g/L	Group 4 AT < 50% FIB < 1.5 g/L
Number (%)	Infectious	932 (56.0)	560 (33.7)	73 (4.4)	99 (5.9)
	Hematopoietic disorder	543 (67.8)	40 (5.0)	194 (24.2)	24 (3.0)
	Other	208 (55.6)	57 (15.2)	68 (18.2)	41 (11.0)
Number, male/female	Infectious	551/381	329/231	33/40	55/44
	Hematopoietic disorder	330/213	26/14	108/86	12/12
	Other	122/86	40/17	29/39	24/17
Age (y)	Infectious	70 (57–79)	72 (63–80)	67 (42–78)	72 (59–80)
	Hematopoietic disorder	59 (37–69)	70 (61.5–75.5)	62 (40–72)	67.5 (49.5–77)
	Other	62 (48–74)	71 (53–78)	56 (26.5–71.5)	39 (0–63)
Hemorrhage, *n* (%)	Infectious	119 (12.8)	66 (11.8)	24 (32.9)	18 (18.2)
	Hematopoietic disorder	168 (30.9)	6 (15.0)	95 (49.0)	3 (12.5)
	Other	48 (23.1)	8 (14.0)	23 (33.8)	16 (39.0)
With renal dysfunction a , *n* (%)	Infectious	145 (15.6)	123 (22.1)	12 (16.4)	24 (24.2)
	Hematopoietic disorder	35 (6.4)	5 (12.5)	2 (1.0)	4 (16.7)
	Other	35 (16.8)	11 (19.3)	8 (11.8)	4 (9.8)
With liver dysfunction b , *n* (%)	Infectious	92 (9.9)	75 (13.4)	16 (21.9)	25 (25.3)
	Hematopoietic disorder	28 (5.2)	11 (27.5)	9 (4.6)	5 (20.8)
	Other	41 (19.7)	12 (21.1)	11 (16.2)	6 (14.6)
SOFA score	Infectious	9.5 (7–12)	11 c (9–14)	10 (7–13)	11 c (9–15)
	Hematopoietic disorder	–	–	–	–
	Other	6 (3–10)	9 d (8–12)	6 (3–10), 23	12 d (10–13)
JAAM DIC score	Infectious	5 (4–6)	6 c (4–7)	6 d (5–7)	6 e (5–7)
	Hematopoietic disorder	–	–	–	–
	Other	4 (3–6)	5 (4–6)	6 c (4–7)	5 (4–7)
ISTH overt- DIC score	Infectious	4 (3–5)	5 c (3–5)	6 d (5–6)	6 e (4–6)
	Hematopoietic disorder	5 (4–5)	5 (4–6)	6 c (5–7)	5 e (4–7)
	Other	4 (2–5)	5 e (3–5)	5 c (4–7)	5 c (4–7)
JMHLW DIC score	Infectious	6 (4–7)	6 e (5–7)	9 c (7–10)	8 c (6–9)
	Hematopoietic disorder	4 (3–4)	3 (3–5)	6 c (5–7)	5 c (4–6)
	Other	5 (4–7)	6 (5–7)	8 c (7–10)	8 c (6–9)
Treatment for DIC					
Respiratory support	Infectious	343 (36.8)	242 (43.2)	37 (50.7)	55 (55.6)
	Hematopoietic disorder	63 (11.6)	11 (27.5)	21 (10.8)	3 (12.5)
	Other	50 (24.0)	25 (43.9)	17 (25.0)	17 (41.5)
CHDF	Infectious	246 (26.4)	177 (31.6)	22 (30.1)	29 (29.3)
	Hematopoietic disorder	22 (4.1)	4 (10.0)	4 (2.1)	5 (20.8)
	Other	34 (16.3)	17 (29.8)	9 (13.2)	10 (24.4)
PMX-DHP	Infectious	104 (11.2)	74 (13.2)	4 (5.5)	11 (11.1)
	Hematopoietic disorder	4 (0.7)	1 (2.5)	0 (0.0)	0 (0.0)
	Other	6 (2.9)	3 (5.3)	0 (0.0)	0 (0.0)
Platelet concentrate transfusion	Infectious	201 (21.6)	126 (22.5)	22 (30.1)	31 (31.1)
	Hematopoietic disorder	224 (41.3)	20 (50.0)	102 (52.6)	9 (37.5)
	Other	45 (21.6)	16 (28.1)	26 (38.2)	18 (43.9)
Fresh frozen plasma transfusion	Infectious	158 (17.0)	131 (23.4)	26 (35.6)	48 (48.5)
	Hematopoietic disorder	68 (12.5)	13 (32.5)	99 (51.0)	9 (37.5)
	Other	47 (22.6)	22 (38.6)	31 (45.6)	26 (63.4)
Heparin derivatives	Infectious	231 (24.8)	134 (23.9)	23 (31.5)	21 (21.2)
	Hematopoietic disorder	129 (23.8)	18 (45.0)	38 (19.6)	7 (29.2)
	Other	55 (26.5)	12 (21.1)	22 (32.4)	8 (19.5)
Antithrombin concentrate	Infectious	469 (50.3)	350 (62.5)	33 (45.2)	66 (66.7)
	Hematopoietic disorder	123 (22.7)	27 (67.5)	24 (12.4)	15 (62.5)
	Other	68 (32.7)	36 (63.2)	25 (36.8)	21 (51.2)
Outcome or resolution rate of DIC					
28-day survival rate	Infectious	76.0%	64.5% c	50.7% c	45.8% c
	Hematopoietic disorder	72.5%	42.5% c	73.7%	41.7% c
	Other	71.0%	61.8%	75.0%	61.0%
JMHLW DIC resolution rate	Infectious	66.8%	58.4%	55.6%	43.2%
	Hematopoietic disorder	59.4%	30.4%	48.3%	31.3%
	Other	42.4%	56.0%	57.9%	45.0%
JAAM DIC resolution rate	Infectious	46.8%	34.9%	22.6%	16.0%
	Hematopoietic disorder	–	–	–	–
	Other	26.0%	35.9%	20.5%	16.0%
ISTH DIC resolution rate	Infectious	74.6%	66.9%	53.2%	46.4%
	Hematopoietic disorder	64.5%	31.8%	60.3%	30.8%
	Other	52.7%	60.0%	45.9%	45.0%

Abbreviations: AT, antithrombin; CHDF, continuous hemodiafiltration; DIC, disseminated intravascular coagulation; FIB, fibrinogen; ISTH, International Society of Thrombosis and Haemostasis; JAAM, Japanese Association for Acute Medicine; JMHLW, Japanese Ministry of Health, Labor and Welfare; PMX-DHP, direct hemoperfusion with polymyxin B immobilized fiber; SOFA, Sequential Organ Failure Assessment.

Note: Data for age and score are shown as medians (25th, 75th percentiles) and numbers. Data for treatment are shown as numbers (%), and data for period are shown as medians (25th, 75th percentiles), numbers. Data for outcome and the resolution rate are shown as percents (numbers/total numbers) and numbers, respectively.

a Serum creatinine > 4.0 mg/dL or on dialysis.

b Total bilirubin > 10 mg/dL, aspartate aminotransferase > 500 IU/L, or alanine aminotransferase > 500 IU/L.

c *p* < 0.001 in comparison with group 1.

d *p* < 0.01 in comparison with group 1.

e *p* < 0.05 in comparison with group 1.

**Fig. 2:**
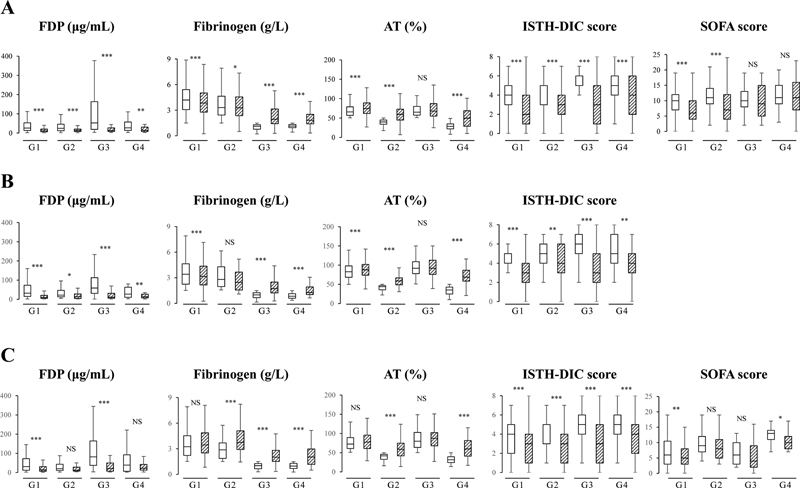
Behavior of hemostatic markers, Sequential Organ Failure Assessment (SOFA) score, and the International Society of Thrombosis Haemostasis (ISTH) disseminated intravascular coagulation (DIC) score after treatment. ( **A** ) Infectious-type DIC; ( **B** ) hematopoietic disorder-type DIC; ( **C** ) other-type DIC; group 1, AT ≥ 50% and FIB ≥ 1.5 g/L; group 2, AT < 50% and FIB ≥ 1.5 g/L; group 3, AT ≥ 50% and FIB < 1.5 g/L; group 4, AT < 50% and FIB < 1.5 g/L; AT, antithrombin; FIB, fibrinogen; open bar, before treatment; diagonal bar, after treatment; FDP, fibrinogen and fibrin degradation products. *** *p* < 0.001; ** *p* < 0.01; * *p* < 0.05; NS, not significant between before and after treatment.

Regarding hemostatic abnormalities, in all types of DIC, in comparison to group 1, plasma fibrinogen levels were significantly lower in groups 3 and 4 (both *p* < 0.001), and plasma AT levels were significantly lower in groups 2 and 4 (both *p* < 0.001) ( Table 3 ). Platelet counts were low in all groups. The PT ratio was significantly higher in groups 2, 3, and 4 than in group 1 (other-type, group 3 *p* < 0.05, all other *p* < 0.001), and FDP and TAT levels were significantly higher in group 3 than in group 1 (FDP, all *p* < 0.001, TAT, infectious-type, other-type *p* < 0.01, hematopoietic disorder-type *p* < 0.001). Regarding hemostatic markers after treatment for DIC ( Fig. 2 ), FDP and fibrinogen levels in all groups of the infectious-type of DIC were significantly improved after treatment (FDP groups 1, 2, 3 *p* < 0.001, group 4 *p* < 0.01, fibrinogen groups 1, 3, 4 *p* < 0.001, group 2 *p* < 0.05). In hematopoietic disorder-type DIC, FDP levels in all groups and fibrinogen levels in groups 1, 3, and 4 were significantly improved after treatment (FDP groups 1, 3 *p* < 0.001, group 4 *p* < 0.01, group 2 *p* < 0.05; fibrinogen groups 1, 3, 4 *p* < 0.001).

**Table 3:** Hemostatic abnormalities of the four groups by the three types of DIC

Parameter	Type of DIC	Group 1 AT ≥ 50% FIB ≥ 1.5 g/L	Group 2 AT < 50% FIB ≥ 1.5 g/L	Group 3 AT ≥ 50% FIB < 1.5 g/L	Group 4 AT < 50% FIB < 1.5 g/L
Platelet count, (× 10 ^3^ /μL)	Infectious	60 (35–94), 930	58 (32–82) c , 558	4.9 (34–81), 73	50 (33–70) c , 99
	Hematopoietic disorder	29 (15–58), 542	27 (12–42), 40	3.15 (17–59), 192	31.5 (21–83), 24
	Other	68 (43–118), 208	58 (34–79), 57	4.5 (28–75) a , 68	56 (33–98), 41
PT ratio	Infectious	1.25 (1.13–1.42), 873	1.37 (1.25–1.59) a , 525	1.52 (1.23–1.97) a , 66	1.74 (1.50–2.19) a , 92
	Hematopoietic disorder	1.21 (1.09–1.34), 506	1.39 (1.25–1.61) a , 40	1.28 (1.17–1.45) a , 185	1.56 (1.3–1.90) a , 20
	Other	1.20 (1.11–1.40), 196	1.41 (1.26–1.77) a , 55	1.29 (1.17–1.56) c , 60	1.69 (1.48–2.18) a , 38
Fibrinogen (g/L)	Infectious	4.18 (3.03–5.44), 932	3.30 (2.40–4.65) a , 560	1.12 (0.812–1.30) a , 73	1.15 (0.93–1.33) a , 99
	Hematopoietic disorder	3.50 (2.27–4.64), 543	2.84 (2.02–4.24), 40	0.996 (0.700–1.24) a , 194	0.844 (0.556–1.18) a , 24
	Other	3.14 (2.29–4.39), 208	2.90 (1.90–3.71), 57	1.01 (0.660–1.27) a , 68	0.99 (0.69–1.31) a , 41
FDP (μg/mL)	Infectious	26.0 (13.3–53), 787	24.5 (12.2–44.6), 462	52.7 (20.6–184) a , 63	27.2 (14.0–57.5), 78
	Hematopoietic disorder	31.0 (15.5–68.0), 502	18.6 (10.0–34.4) b , 40	60.0 (33.9–125) a , 182	29.4 (12.3–52.9), 21
	Other	26.2 (11.4–65.2), 168	19.8 (11.1–38.6), 49	81.4 (37.3–162) a , 57	29.2 (7.7–86.3), 32
Antithrombin (%)	Infectious	66.0 (57.0–78.1), 932	40.0 (33.7–45.0) a , 560	65.2 (58.0–77.6), 73	30.0 (22.0–38.0) a , 99
	Hematopoietic disorder	84.1 (70.0–99.7), 543	44.1 (39.4–47.4) a , 40	92.5 (79.0–111) a , 194	35.2 (25.0–42.8) a , 24
	Other	76.0 (61.1–90.0), 208	41.0 (35.3–46.2) a , 57	84.2 (64.3–102), 68	34.0 (27.8–40.1) a , 41
TAT (ng/mL)	Infectious	13.7 (6.7–25.2), 318	11.2 (6.6–20.5), 169	30.65 (11.6–60.5) b , 26	15.8 (7.2–21.1), 28
	Hematopoietic disorder	15.6 (8.5–27.0), 199	14.5 (6.4–23.1), 17	37.4 (19.9–62.9) a , 68	21.2 (20.1–51.0), 5
	Other	14.7 (4.75–29.4), 64	16.9 (8.0–27.1), 12	49.4 (11.0–78.2) b , 30	39.5 (8.2–141), 12
PIC (μg/mL)	Infectious	1.8 (0.9–3.7), 238	1.4 (0.8–2.4), 130	2.9 (0.9–4.6), 21	1.9 (0.8–3.8), 21
	Hematopoietic disorder	3.8 (2.2–7.7), 187	1.5 (1.0–3.3) c , 13	12.4 (6.1–16.2) a , 70	1.25 (1.1–2.4) c , 6
	Other	2.0 (1.0–7.2), 50	0.8 (0.4–1.4) c , 7	6.1 (1.2–9.9), 29	2.3 (0.5–4.8), 12
CRP (mg/dL)	Infectious	17.0 (10.0–24.03), 903	17.49 (11.52–24.1), 541	2.9 (0.81–6.05) a , 69	5.96 (2.47–9.22) a , 91
	Hematopoietic disorder	7.8 (2.89–16.51), 501	15.62 (7.6–21.4) b , 35	1.06 (0.27–2.83) a , 173	5.78 (2.0–8.94), 23
	Other	7.86 (2.76–15.49), 195	10.94 (4.31–17.2), 54	0.72 (0.18–2.7) a , 63	0.47 (0.06–1.64) a , 35

Abbreviations: AT, antithrombin; CRP, C-reactive protein; DIC, disseminated intravascular coagulation; FDP, fibrin degradation product; FIB, fibrinogen; PIC, plasmin inhibitor complex; PT, prothrombin time; TAT, thrombin-antithrombin complex.

Note: Data for age and score are shown as medians (25th, 75th percentiles) and numbers, respectively.

a *p* < 0.001 in comparison with group 1.

b *p* < 0.01 in comparison with group 1.

c *p* < 0.05 in comparison with group 1.

**Fig. 3:**
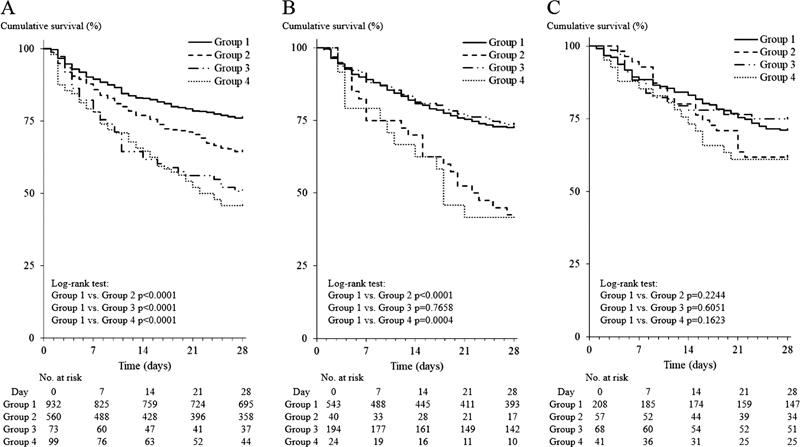
Survival curves in patients in groups 1, 2, 3, and 4. ( **A** ) Infectious-type disseminated intravascular coagulation (DIC); ( **B** ) hematopoietic disorder-type DIC; ( **C** ) other-type DIC; group 1, AT ≥ 50% and FIB ≥ 1.5 g/L; group 2, AT < 50% and FIB ≥ 1.5 g/L; group 3, AT ≥ 50% and FIB < 1.5 g/L; group 4, AT < 50% and FIB < 1.5 g/L. AT, antithrombin; FIB, fibrinogen.

**Fig. 4:**
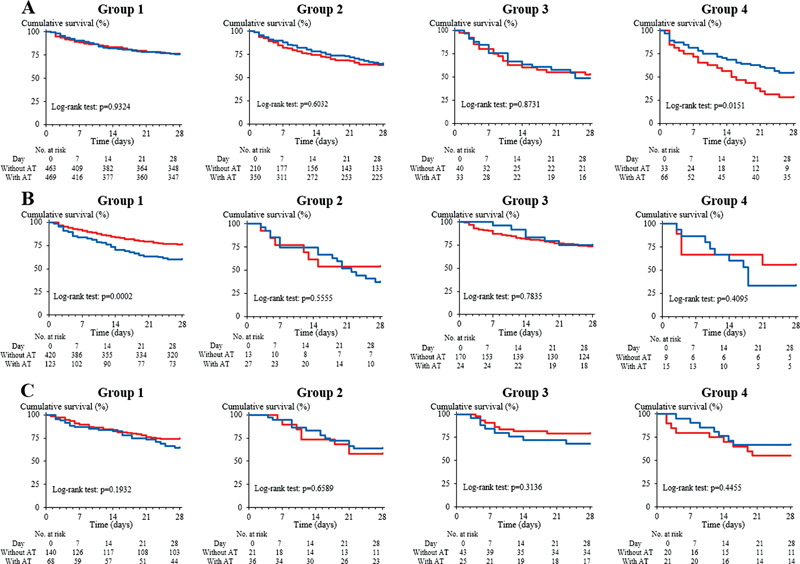
Survival curves in patients treated with antithrombin concentrate (blue line) or without antithrombin concentrate (red line). ( **A** ) infectious-type disseminated intravascular coagulation (DIC); ( **B** ) hematopoietic disorder-type DIC; ( **C** ) other-type DIC; group 1, AT ≥ 50% and FIB ≥ 1.5 g/L; group 2, AT < 50% and FIB ≥ 1.5 g/L; group 3, AT ≥ 50% and FIB < 1.5 g/L; group 4, AT < 50% and FIB < 1.5 g/L. AT, antithrombin; FIB, fibrinogen.

The survival curves showed that the survival rate was significantly lower in groups 2, 3, and 4 (all *p* < 0.001) than in group 1 in infectious-type DIC and in groups 2 and 4 (both *p* < 0.001) than in group 1 in hematopoietic disorder-type DIC. There was no significant difference in the survival curves among groups 1 to 4 of other-type DIC ( Fig. 3 ). Regarding combination therapy with TM-α and AT, the survival curve was significantly higher ( *p* < 0.05) only in group 4 patients with infectious-type DIC treated with combination therapy than in those treated without combination therapy. The survival curve was significantly lower ( *p* < 0.001) only in group 1 patients with hematopoietic disorder-type DIC treated with combination therapy than in those treated without combination therapy. There were no significant differences in the survival curves between combination and noncombination therapy for groups 1 to 3 of infectious-type, groups 2 to 4 of hematopoietic disorder-type DIC, and groups 1 to 4 of other-type DIC ( Fig. 4 ).

The 28-day survival rate and resolution rates from DIC decreased in order of groups 1, 2, 3, and 4 in infectious-type DIC, and they decreased in order of groups 1, 3, 2, and 4 in hematological malignancy. The 28-day survival rate and resolution rates from DIC were generally low in other-type DIC ( Table 2 ). The ISTH overt-DIC score in all groups of three DIC types was significantly lower after treatment than before treatment (infectious-type, other-type, all *p* < 0.001, hematopoietic disorder-type groups 1, 3 *p* < 0.001, groups 2, 4 *p* < 0.01) ( Fig. 2 ). The SOFA score in groups 3 and 4 of infectious-type DIC and in groups 2 and 3 of other-type DIC was not significantly lower after treatment than before treatment.

## Discussion

DIC is a unique condition in which thrombogenesis and a bleeding tendency coexist. This unique feature is often complicated by severe organ failure and bleeding that leads to poor outcomes. 22 23 Therefore, although clinical evidence has not been sufficient, anticoagulant therapy was proposed for the treatment of DIC. Another unique aspect of DIC is the wide diversity in its pathophysiology, as well as phenotype, depending on the underlying diseases. Consequently, an individual approach is required for each DIC type. The cornerstone of an anticoagulation study was the success in the treatment of severe sepsis by activated protein C, 25 but this study did not target DIC, and the subsequent studies could not reproduce the results. 20 Meanwhile, recent studies showed a tight connection between coagulation and inflammation in sepsis, and the studies on AT and TM-α showed some beneficial effects of anticoagulation on severe sepsis. 19 21 26 27 Taken together, the effects of anticoagulant therapy using AT or TM-α may be beneficial for sepsis, but the effects appear to be limited to patients with DIC.

The prognosis of DIC remains poor, 1 19 25 and decreases in coagulation and anticoagulant factors can predict poor outcomes. 22 23 28 Severe AT deficiency is known to be associated with a high risk of organ failure and death in patients with severe sepsis. 22 29 Together with the activated coagulation, decreased fibrinolysis due to excess production of plasminogen activator inhibitor 1 accelerates the microthrombosis and poor circulation in sepsis. 22 As a countermeasure, supplementation with AT in DIC patients with serum AT activity ≤ 70% is approved in Japan, and previous studies have demonstrated that AT activity ≤ 50% could predict a poor outcome. 22 29 Similarly, hypofibrinogenemia is also helpful to evaluate the severity of sepsis. 23 Hypofibrinogenemia reflects the consumptive coagulopathy that leads to the hemostatic disorder. It is noteworthy that the true fibrinogen level is lower than the measured fibrinogen activity in DIC, since the fibrinogen level is usually measured using a clotting assay despite the presence of a hypercoagulable state. Although prolonged PT and thrombocytopenia are widely accepted as prognostic markers, hypofibrinogenemia is also expected to be predictive. The strongest predictor of poor outcomes was hypofibrinogenemia in DIC associated with infection and decreased AT activity in DIC associated with hematological malignancy. Accordingly, we have reported that conditions with AT activity < 50% and fibrinogen levels < 1.5 g/L were strongly associated with poor outcomes in DIC. 28 The present study also confirmed the usefulness of evaluating the severity of DIC using AT activity and fibrinogen.

Evaluation of prognosis and severity by adding AT to TM-α was helpful for selecting the target of intensive anticoagulation. Kienast et al 26 have reported that treatment with high-dose AT without concomitant heparin resulted in a significant mortality reduction in septic patients with DIC, suggesting that AT could be a beneficial treatment for DIC associated with sepsis. In addition, small observational studies also reported the potential efficacy of combination therapy with AT and TM-α. 30 Since the present study also showed the association between combination therapy and improved survival in septic patients with AT < 50% and fibrinogen < 1.5 g/L, we think that these patients can be the optimal target for future clinical trials. A hyperfibrinolytic state is considered in hematological malignancy patients with AT activity ≥ 50% and a fibrinogen level < 1.5 g/L, suggesting that not only anticoagulant therapy, but also antifibrinolytic therapy such as tranexamic acid may be required in this type of DIC.

There are some limitations to this study. First, the data set was obtained from the PMS of TM-α, and all patients were treated with TM-α. Therefore, only the additive effects of AT are discussed. Second, the sample size had a large variation because this was a post hoc study. Third, the timing of treatment was not restricted. Since early initiation of treatment is advocated, a study that controls treatment timing is warranted.

In conclusion, the present study used a large PMS database that showed the association between improved outcomes and intensive anticoagulation by adding AT to TM-α in sepsis-based DIC patients with AT activity < 50% and fibrinogen levels < 1.5 g/L. The study result provides important information for future trials. However, low AT or low fibrinogen levels are considered to indicate a poor outcome, and both low-AT and low-fibrinogen levels suggest very poor outcomes; all patients with poor outcomes did not always have low AT or low fibrinogen.

## Notes

**What is known about this topic?**

- DIC can be classified as infectious-type DIC, hematopoietic disorder-type DIC, or other-type DIC.
- DIC patients are poor outcome.
- DIC patients with decreased antithrombin or fibrinogen levels are poor outcome.
- Antithrombin or thrombomodulin therapy is useful for DIC.
- The usefulness of combination therapy with antithrombin and thrombomodulin are still not established.

**What does this paper add?**

- DIC patients with low-antithrombin and low-fibrinogen risk poor outcomes.
- These patients due to infection can be the target of combination therapy with antithrombin and thrombomodulin.
